# How Many People Could Use an SSVEP BCI?

**DOI:** 10.3389/fnins.2012.00169

**Published:** 2012-11-19

**Authors:** Christoph Guger, Brendan Z. Allison, Bernhard Großwindhager, Robert Prückl, Christoph Hintermüller, Christoph Kapeller, Markus Bruckner, Gunther Krausz, Günter Edlinger

**Affiliations:** ^1^g.tec Medical Engineering GmbH, Guger Technologies OGGraz, Styria, Austria; ^2^Department of Cognitive Science, University of California at San DiegoLa Jolla, CA, USA

**Keywords:** brain-computer interface, brain-machine interface, steady-state visual evoked potential, SSVEP, motor imagery

## Abstract

Brain-computer interfaces (BCI) are communication systems that allow people to send messages or commands without movement. BCIs rely on different types of signals in the electroencephalogram (EEG), typically P300s, steady-state visually evoked potentials (SSVEP), or event-related desynchronization. Early BCI systems were often evaluated with a selected group of subjects. Also, many articles do not mention data from subjects who performed poorly. These and other factors have made it difficult to estimate how many people could use different BCIs. The present study explored how many subjects could use an SSVEP BCI. We recorded data from 53 subjects while they participated in 1–4 runs that were each 4 min long. During these runs, the subjects focused on one of four LEDs that each flickered at a different frequency. The eight channel EEG data were analyzed with a minimum energy parameter estimation algorithm and classified with linear discriminant analysis into one of the four classes. Online results showed that SSVEP BCIs could provide effective communication for all 53 subjects, resulting in a grand average accuracy of 95.5%. About 96.2% of the subjects reached an accuracy above 80%, and nobody was below 60%. This study showed that SSVEP based BCI systems can reach very high accuracies after only a very short training period. The SSVEP approach worked for all participating subjects, who attained accuracy well above chance level. This is important because it shows that SSVEP BCIs could provide communication for some users when other approaches might not work for them.

## Introduction

Brain-computer interfaces (BCIs) are communication systems in which direct measures of the user’s brain activity are translated into command and control signals. Most modern BCIs rely on one of three types of signals recorded from the electroencephalogram (EEG): event-related desynchronization/synchronization (ERD/ERS), P300, or steady-state visual evoked potential (SSVEP; Middendorf et al., 2000; Wolpaw et al., 2002; Gao et al., 2003; Guger et al., 2003, 2009, 2012a; Sellers et al., 2006; Pfurtscheller et al., 2010; Ortner et al., 2011). ERD BCIs usually require subjects to imagine movement, while subjects who use P300 and SSVEP BCIs usually must pay attention to a specific visual target that flashes (in a P300 BCI) or oscillates (in an SSVEP BCI). The first patient that typed a letter with a BCI was using slow waves, but this approach is seldom used nowadays because it requires extensive training and is less accurate and robust than other approaches (Birbaumer et al., 1999). BCIs based on invasive methods such as the Electrocorticogram (ECoG) or action potentials have also been described (Hochberg et al., 2006; Leuthardt et al., 2011).

Event-related desynchronization BCIs have many appealing features. For example, they usually require subjects to imagine movements, which can be very intuitive, natural control signals for many BCI tasks (such as moving a wheelchair or an avatar). They also do not require external stimuli to generate the brain activity needed for control, although some external stimulation is necessary in any BCI to provide feedback (Wolpaw et al., 2002; Pfurtscheller et al., 2010). However, ERD BCIs may require training subjects to attain adequate control and find a suitable type of imagery (such as playing tennis or weight lifting), require attention to this imagery, and may encounter greater problems with “illiteracy” than other BCIs (Guger et al., 2003, 2009; Allison and Neuper, 2010; Allison et al., 2010; Vidaurre et al., 2011). Motor imagery based BCI are also gaining attention in stroke rehabilitation and other applications.

In a previous study, we tested a motor imagery based BCI system with 99 subjects visiting an exhibition in Graz (Guger et al., 2003). The subjects were trained for 6 min to imagine left or right hand movement for a few seconds (20 times each) to produce ERD and ERS changes. The BCI system was then trained on the individual EEG data for a subsequent session with visual feedback of cursor movement. The subjects were able to move the cursor to the left or right side of the computer screen. About 6.2% of the subjects were able to learn this control with >90% accuracy in this short training session. About 93.3% showed a control above 59% accuracy (50% corresponds to random classification).

P300-based BCIs present many choices on a computer screen that are highlighted randomly (Sellers et al., 2006; Guger et al., 2009, 2012a). The subject must focus on the target that he or she wants to select. Each target flash produces a P300 response in the EEG, which is recognized by the BCI. Normally, every item is highlighted several times to improve the signal-to-noise ratio, and hence several seconds are required to identify each character. P300 systems are well suited for item selection applications such as spelling (Mason et al., 2007).

The P300-based BCI can achieve high accuracy after only 5 min of training (Guger et al., 2009, 2012a). About 72.8% of the subjects reached 100% accuracy with the row-column speller. Moreover, these results show accuracy levels similar to those of other studies that have used much more training data (Serby et al., 2005; Krusienski et al., 2006; Sellers et al., 2006, 2008; Nijboer et al., 2008).

Steady-state visually evoked potentials based BCI systems use multiple visual stimuli (such as LEDs or boxes on a computer screen) that flicker at different frequencies (Friman et al., 2007; Martinez et al., 2007; Bin et al., 2009). The subject has to focus on the item he or she wants to select, which elicits the stimulation frequency in the EEG. Typically, 0.5–3 s of data are analyzed and used to perform the selection before the window is moved to the next time point. The resulting real-time classification can be used, e.g., to steer a robotic device (Grave de Peralta Menendez et al., 2009). About 42% of male and 65% of female users reached 100% accuracy in a group study (Allison et al., 2010).

The P300 BCI generates a trigger signal for every flashing item and therefore it is termed to be a synchronous BCI system. Both the motor imagery and the SSVEP do not need necessarily a trigger signal and do the classification on the fly, and are therefore termed asynchronous BCI systems. But motor imagery and SSVEP based BCIs also perform much worse if the BCI system should discriminate when the subject is doing nothing, versus performing motor imagery or attending to the SSVEP stimulus. This is known as the zero-class problem, and is an underappreciated issue in real-world BCI systems (Huggins et al., 2011). BCI systems that address this problem could work with trigger information, which tells the person when to perform the necessary mental tasks (Guger et al., 2001).

Based on experience with the motor imagery and P300 group studies (Guger et al., 2003, 2009, 2012a), we sought to replicate the design using a SSVEP based BCI. One prior effort (Allison et al., 2010) tried to explore SSVEP universality across many subjects. But the study reported only results of 65 out of 106 subjects. This is an important difference from the study presented here, which reports all of the participating subjects. The principal goal of this study was to estimate how many people could use an SSVEP BCI based on data from a large number of subjects.

## Materials and Methods

### Participants

Fifty-three people volunteered for this study (18 female; age range 18–73, mean age 29 ± 13). All subjects had normal or corrected to normal vision and provided informed consent and were recruited through word-of-mouth or through flyers posted at Johannes Kepler University or Fachhochschule Linz. All of the people aged 18 or older who wanted to participate were run as subjects, and data from all subjects who participated are reported here. All of the subjects had never used a BCI, and did not report any mental or physical disability. Ethical approval was obtained from the Medical University of Graz.

### Experimental procedure

**Figure 1:**
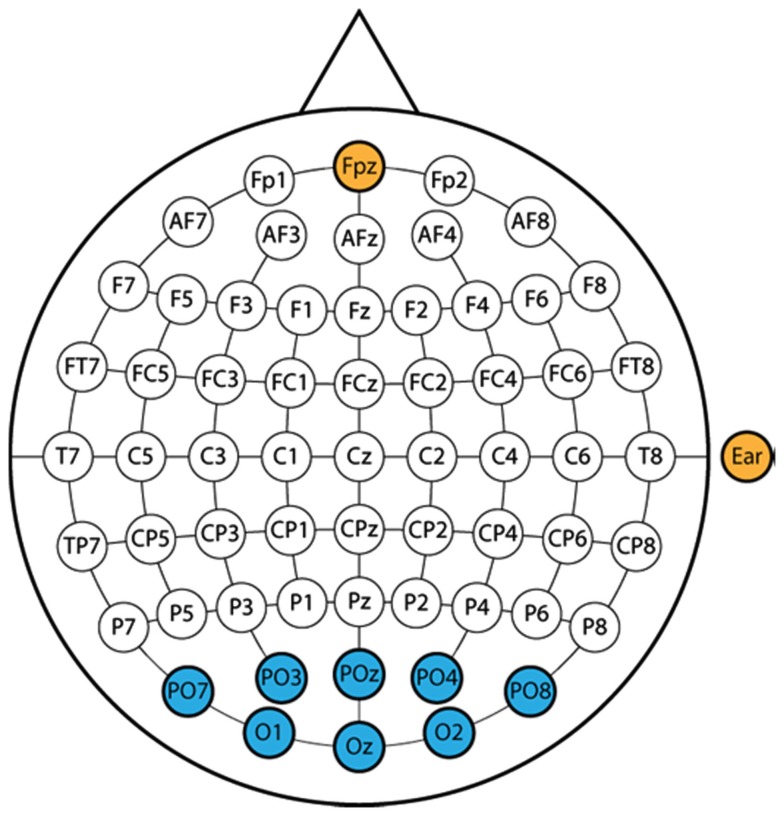
**The eight recording sites are shown in blue**. The two yellow sites reflect a ground electrode at FPz and a reference electrode on the right earlobe. All electrodes except the ground electrode are active electrodes to reduce preparation time, reduce noise, improve the signal-to-noise ratio, and eliminate the need for skin abrasion.

Each subject was prepared for recording using gold plated active electrodes. These electrodes require a small amount of electrode gel, and do not require skin abrasion. Figure 1 shows the electrode montage used in this study. Data were recorded from eight posterior electrode sites positioned according to the international 10–20 electrode system, with a reference electrode on the right earlobe and a ground electrode over site FPz. Electrode preparation took about 2 min. Data were sent to a g.USBamp amplifier sampling at 256 Hz, with a bandpass filter of 0.5–30 Hz and a notch filter at 50 Hz. The device performed oversampling at 2.4 GHz to increase the signal-to-noise ratio.

**Figure 2:**
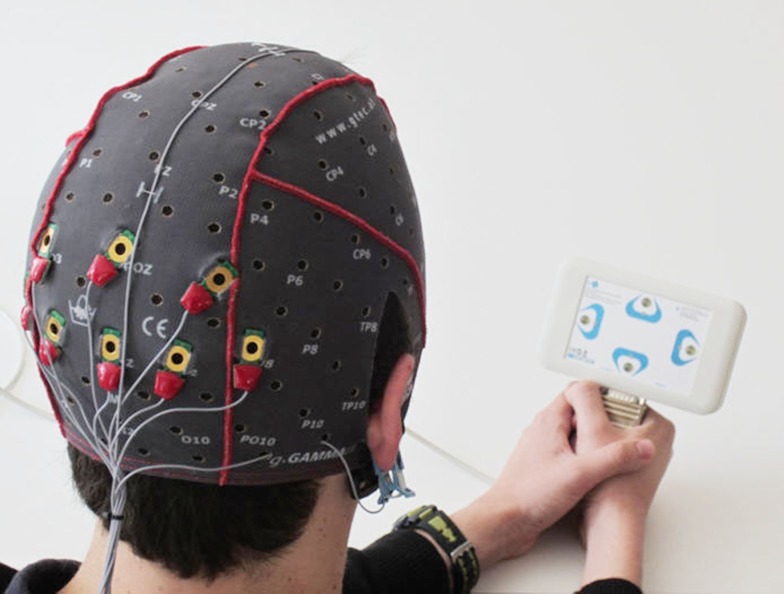
**A subject is prepared for recording and holds the SSVEP box used to present stimuli**. The top LED is flickering at 10 Hz, corresponding to upward movement. The right, bottom, and left LEDs flickered at 11, 12, and 13 Hz, respectively.

Once the electrode cap was in place, and the experimenter visually inspected the resulting EEG data, each subject participated in one training run. Subjects viewed a SSVEP box, which has four stimulation LEDs positioned on the top, right, bottom, and left (See Figure 2). The run began with a 10 s delay, and each trial began with a 3 s pause. Next, the four LEDs began to oscillate at 10 Hz (top box), 11 Hz (right box), 12 Hz (bottom box), or 13 Hz (left box). Simultaneously, a small green light appeared about 2 mm from one of the four LEDs, which cued the subjects to focus on that LED.

Subjects were seated in front of the SSVEP box shown in Figure 2 and were asked to focus on the target LED for 7 s, after which the trial ended and the lights on the SSVEP box turned off. The top LED was designated as the target for the first five trials, followed by five trials for the right LED, then the bottom LED, then the left LED. After these 20 trials ended, the classifier was trained on the resulting data while subjects took a short break. This training procedure (recording data and training the classifier) took about 5 min. This classifier was used to classify the EEG data in real-time for the following run and to present the classification result. This run was identical to the training run from the subject’s perspective. After this run, the online classification result was used to calculate the accuracy of the run and the experimenter told the subject of the resulting peak accuracy after each run. Then, subjects could choose to participate in another run. Subjects were allowed up to four online runs with the cued stimuli. After completing these runs, subjects could choose to use the system for “free spelling” runs, which were not recorded nor discussed further.

### Hardware and software

**Figure 3:**
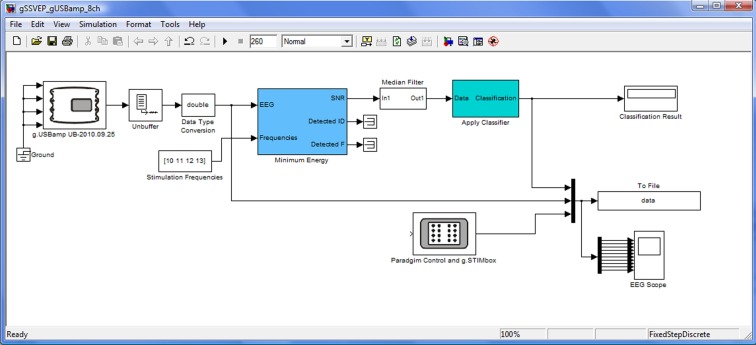
**Real-time simulink model running the SSVEP experiment**.

All experiments were managed by g.BCIsys, which uses Simulink as a rapid prototyping platform to run real-time experiments (Guger et al., 2001). Figure 3 shows the real-time Simulink model that controlled the data acquisition, feature extraction, classification, paradigm, data visualization, and storage. The g.USBamp block reads the data of eight EEG channels into the Simulink model at 256 Hz in blocks of eight samples. Data were then unbuffered to update the model sample by sample and converted to double precision for high precision for the signal processing steps. Then the minimum energy algorithm (Friman et al., 2007) optimized the signal-to-noise ratio for each of the stimulating frequencies (10, 11, 12, 13 Hz) and all eight EEG channels. This algorithm used a Levinson AR Model (order 7) that used the preceding 768 sample points (3 s). Every 200 ms, the Simulink model updated the features. The resulting features were smoothed with a median filter before a linear discriminant analysis (LDA) classifier was used for pattern classification (Guger et al., 2001). Finally, the target selected by the classifier was presented in a display block (Classification Result) within the Simulink model. The Paradigm Control and g.STIMbox block is controlling the experimental procedure and the LEDs with a μC board. The To File block stores the EEG data, the classifier output and the ID of the target LED (1–4) in MATLAB format for training and off-line analysis, and these data can also be visualized in a Scope for data inspection.

## Results

**Figure 4:**
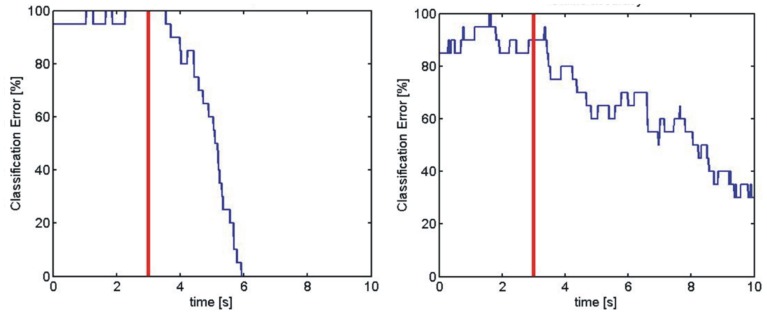
**These two panels present online errors from two subjects**. The left panel is from one of the best subjects, and the right panel is from one of the worst subjects. The red vertical line indicates cue onset, and the blue line presents the error rate throughout the trial.

The classification error calculated from the online classification result for two subjects is shown in Figure 4. The Figure presents the error rate from the best and worst individual subjects. In addition to attaining lower peak error, the best performer also reduced error more quickly after cue onset, and the error stayed at zero until the end of the trial, whereas the worst performer seemed to continue improving throughout the trial (Guger et al., 2012b).

Table 1 summarizes the results of the current study for all 53 subjects. Each cell presents the highest online peak accuracy the subject attained, grouped within different ranges. Hence, a result of 100% accuracy did not necessarily reflect perfect performance throughout a trial. Fifty-three subjects completed the first run with feedback, attaining a mean accuracy of 87.9%. Twenty-two subjects reached perfect accuracy, while seven subjects were below 60%. Fourteen subjects also performed a second run with feedback, which increased the mean accuracy to 92.9%. Seven subjects completed a third feedback run, and two subjects completed a fourth feedback run, yielding a final mean accuracy of 95.5%. Nobody was below 60% accuracy after their last run. Notably, if subjects completed two or more runs, then their performance from the last run is used, which is not necessarily the best. Ultimately, 50.9% of subjects reached a perfect accuracy of 100%, and only 3.8% were between 60 and 79%.

**Table 1:** **This table summarizes subjects’ performance**.

Accuracy (%)	Number of subjects performing at specified accuracy				Percentage of people after training
	Run 1	Run 2	Run 3	Run 4	
100	22	25	27	27	50.9
90–99	14	19	19	19	35.8
80–89	7	4	5	5	9.4
70–79	2	1	0	1	1.9
60–69	1	2	1	1	1.9
50–59	4	1	0	0	0.0
40–49	3	0	1	0	0.0
0–39	0	1	0	0	0.0
Mean accuracy	87.9	92.9	95.0	95.5	
	*N* = 53	*N* = 53 with 14 new	*N* = 53 with 7 new	*N* = 53 with 2 new	

*The accuracies presented in each cell reflect the highest accuracy the subject attained. Since most subjects performed only one run, most of the results reflect performance after one run. The bottom row reflects the number of subjects who participated in at least the specified number of runs. For example, 53 subjects participated in at least one run, while seven subjects participated in at least three runs*.

Table 2 summarizes the results from this study and two previous studies (Guger et al., 2003, 2009) that each assessed universality with one of the major non-invasive BCI approaches. The table lists different parameters to help compare different BCI systems.

**Table 2:** **This table compares performance across three studies that assessed universality within the three major non-invasive BCI approaches using a large number of subjects**.

	Motor imagery [Guger 03] *N* = 99	P300 speller [Guger 09] *N* = 81	SSVEP *N* = 53
Population with 90–100% accuracy	6.2%	72.8%	86.7%
Population below 80%	80.8%	11.1%	3.8%
Training time	6 min	5 min	4–16 min
Number of electrodes	5	10	10
Random classification accuracy	1/2	1/36	1/4
Decision time for one selection	4 s	About 45 s with 15 flashes	7 s

## Discussion

This study yielded a grand average accuracy of 95.5% and showed that SSVEP BCIs can provide communication for all healthy subjects that participated. Importantly, this is an online result based on a classifier that was calculated from a previous run within the same session. A similar mean accuracy of 95.78% was found in a spelling task (Allison et al., 2010), but that study did not report all subjects, whereas the present study did not exclude any subjects.

In this study 10, 11, 12, and 13 Hz were used as stimulation frequencies without subject specific adaptations. These frequencies worked well even though the frequencies are only separated by 1 Hz and could overlap with strong alpha activity. Optimizing these stimulation frequencies for every subject could yield further improvements. A time window of 3 s was used in this study for the minimum energy estimation before the time window was shifted forward for the next estimation. The 3 s time window yields high accuracies, but a shorter time window could make the BCI faster. The proper selection of the time window also depends on each subject’s abilities and preferences, as well as the application that the BCI controls. Shorter windows might be appropriate for spelling applications, while longer applications are important for control applications where robustness is important.

A comparison of the three approaches is also of interest. SSVEP and P300 BCIs showed a higher population with 90–100% accuracy, and a smaller population below 80% accuracy, compared to motor imagery BCIs (Guger et al., 2003, 2009, 2012a). All of these the studies entailed comparable training time, but the P300 and SSVEP studies used more EEG electrodes. The table suggests that SSVEP BCIs perform well by comparison. However, there are many other differences between these three BCI approaches, and other ways to compare BCIs. This table is certainly not intended to reflect the performance of any ERD, P300, or SSVEP BCI – only the specific BCI systems used in each study. In particular, performance with an ERD BCI that used more modern signal processing and other algorithms would probably be better than the 2003 paper.

The P300, SSVEP, and motor imagery group study show that screening setups could help identify the best BCI for each user within a short time by comparing different approaches. Moreover, subjects who have trouble with one BCI might have better results with another one. The comparison showed that the P300 and SSVEP BCIs might be viable alternatives for people who perform poorly with ERD BCIs. However, this hypothesis needs to be confirmed with a broader within-subjects study in which each participant uses all three approaches.

While the main goal was to assess universality, we also explored the differences between good and bad performers. Visual inspection of the data showed that subjects who attained 100% accuracy often maintained this performance throughout the remainder of the trial. However, this did not always happen, and the performance fluctuations within trials merit further study (Daly et al., 2012; Guger et al., 2012b). Subjects who did not attain high accuracy also needed more time after each cue before performance began to improve. Poor performers also tended to improve throughout the trial. Taken together, these results suggest that some people perform poorly partly because they need more time to develop and/or implement attention strategies needed for effective control. This raises the possibility that poor performers may attain better accuracy with longer trials, particularly if the trials only consider data several seconds after cue onset.

The overall mean accuracy increased with training from 87.9 to 95.5%. The largest increase occurred between the first and second runs, partly because 15 out of 53 subjects performed a second run. Also, some subjects misunderstood the task in the first round, or did not focus on the target appropriately. One drawback of the flexible paradigm in this study, which allowed subjects to choose whether to participate in additional training, is that detailed statistical analyses are not feasible. Moreover, seven subjects who were initially unable to use the BCI effectively became proficient SSVEP BCI users with (at most) only 12 min of additional training. Table 2 shows that seven subjects could not attain 60% accuracy in their first run, yet all subjects did so after training. This result suggests that SSVEP BCIs could be useful to even more people that previously recognized, and that the effects of short-term training should be further studied.

The present results might create the misleading impression that everyone could use an SSVEP BCI. None of the subjects were “illiterate” in the present study, a phenomenon that has also been reported in some other studies (e.g., Martinez et al., 2007). However, other studies have reported that at least one healthy subject could not use an SSVEP BCI (for review, see Allison and Neuper, 2010). Another study (Allison et al., 2010) reported that some people could not use the SSVEP BCI described in that system. Moreover, since BCI performance was not reported for many subjects in that study, the present study was necessary to evaluate SSVEP BCI “universality” across all people who participated. A big advantage of the current study is that the experimental runs were very short and lasted only 4 min until the accuracy was calculated and reported to the subject. Therefore, subjects were informed regularly in short intervals about their performance, and subjects became quickly confident about their SSVEP skills. This point should also be considered for other experimental setups.

Results also suggested that even brief training could improve performance. This improvement is especially interesting because subjects did not receive feedback except for the experimenter informing them of their performance after each run. This is important because training without feedback is not distracting subjects and could yield higher accuracies. After successful training, the BCI system can be switched to feedback mode, which may improve control because of the better calibration data. This result could mean that subjects who cannot initially control an SSVEP BCI could improve with training, much like people who use an ERD BCI (McFarland et al., 2010; Vidaurre et al., 2011).

## Conclusion

The paper showed that all of the subjects who participated can achieve acceptable accuracies with SSVEP based BCIs after a very short training interval, and most subjects could attain 100% peak accuracy. The grand average accuracy is higher than with motor imagery and P300-based BCI systems. Even short training runs can improve performance, which has not been previously appreciated in SSVEP BCI systems. Additional studies are needed to develop and describe training procedures to further improve the performance, customize each SSVEP BCI to individual users, and address the zero-class problem by extending the current methods to identify when users do not want to select anything.

## Conflict of Interest Statement

All of the authors (except the second author) are full-time employes of g.tec medical engineering GmbH, Guger Technologies OG, and the first and last authors are its co-CEOs.
